# Exploring individual character traits and behaviours of clinical academic allied health professionals: a qualitative study

**DOI:** 10.1186/s12913-023-10044-2

**Published:** 2023-09-23

**Authors:** Elizabeth King, Terry Cordrey, Owen Gustafson

**Affiliations:** 1 Oxford Allied Health Professions Research and Innovation Unit, Oxford University Hospitals NHS Foundation Trust, Oxford, OX3 9DU UK; 2 Centre for Movement, Occupational, and Rehabilitation Sciences, Oxford Brookes University, Oxford, OX3 0BP UK

**Keywords:** Characteristics, Behaviour, Clinical academic, Allied health professions, Research, Qualitative, Semi-structured interviews

## Abstract

**Background:**

Clinical academic allied health professionals can positively impact patient care, organisational performance, and local research culture. Despite a previous national drive to increase these roles, they remain low in number with no clear strategy for growth. Reported barriers to this growth cite organisational and economic factors with little recognition of the challenges posed to individuals. There is a lack of research to help allied health professionals understand the personal challenges of clinical academic training and practice. The aim of this study is to explore the character traits and behaviours of clinical academic allied health professionals to understand the individual attributes and strategies taken to pursue a career in this field.

**Methods:**

A semi-structured interview study design was used to collect data from aspiring and established clinical academic allied health professionals. Participants were recruited voluntarily through social media advertisement (aspiring) and purposively through direct email invitation (established). Participants were asked about their experience of pursuing a clinical academic career. The interviews were conducted virtually using Zoom and were audio recorded. The data were transcribed verbatim prior to reflexive thematic analysis. Informed consent was gained prior to data collection and the study was approved by the university’s research ethics committee.

**Results:**

Twenty participants from six allied health professions were interviewed. We developed five themes: risk and reward, don’t wait to be invited, shifting motivations, research is a team sport, and staying the course. Clinical academic allied health professionals demonstrated traits including inquisitiveness, intuition, motivation, and resilience. The source of their motivation was rooted in improving clinical services, conducting research, and personal achievement.

**Conclusion:**

Clinical academic allied health professionals describe personal traits of high inquisitiveness, opportunism, motivation, and determination in pursuing their career ambitions. The tolerance of rejection, failure, and risk was considered important and viewed as an essential source for learning and professional development. Future research should concentrate on ways to reduce the over-reliance on individual strength of character to succeed in this field and explore programmes to increase the preparedness and support for clinical academics from these professions.

**Supplementary Information:**

The online version contains supplementary material available at 10.1186/s12913-023-10044-2.

## Background

Clinical academic allied health professionals (AHPs) are registered clinicians working in clinical practice whilst conducting research within the same role [1]. These posts are reported as beneficial to patient outcomes, healthcare team performance, and local research culture [2]. Despite this, AHPs are under-represented as clinical academics compared to other healthcare professions, such as medicine, with fewer than one in one hundred AHPs funded to undertake research within their clinical role [3, 4]. This low number puts at risk the proliferation of knowledge generation, evidence-informed practice, and clinical innovation to address the real-world challenges faced by these professions and the wider health and care sectors [5]. Findings from several cross-sectional surveys on research capacity and culture show that AHPs perceive themselves with low capability in the necessary skills to conduct original research [6–8]. This illustrates the considerable knowledge and skills gap faced by AHPs trying to enter the highly competitive world of clinical academic training and practice from their clinical role [9].

The need to improve AHP research capacity through clinical academic career development in England has long been recognised. Over a decade ago, a national policy commitment set out aspirations for the AHP workforce to be “…instrumental in ensuring diffusion and spread of best practice and innovation” [10]. The mechanism to help realise this ambition was levied through the National Institute of Health Research’s integrated clinical academic programme (NIHR ICAP). This competitive programme funds individuals to undertake clinical academic training fellowships ranging from entry-level internships to advanced post-doctoral programmes [11]. The benefits of the NIHR ICAP programme to individual award holders are widely reported, but the extent to which this policy and the NIHR ICAP programme have increased AHP clinical academic capacity is currently unknown [12]. Programme evaluations that include the AHP workforce have been limited to participant experience and the equality of award dissemination rather than measures of clinical academic capacity and impact [13].

Key barriers to advancing this agenda are almost exclusively reported as policy, economic and organisational factors with impedance cited as a lack of funding, limited career options, and poor organisational support [14]. Whilst addressing these issues is fundamentally important, doing so without recognising the challenges faced at an individual level risks undermining future improvement strategies [15]. AHPs are motivated to pursue clinical academic careers but report a lack of role modelling, preparedness, high levels of frustration, and challenges to personal resilience [16]. Without the right support and preparation, individuals are vulnerable to work-related stress, working excessive unpaid hours, and attrition from clinical academic training programmes and roles [17]. To our knowledge, no previous research has investigated the experience of AHPs in relation to the personal characteristics, behaviours and strategies to understand what might be required to be a clinical academic. The primary aim of this study is to address this knowledge gap by exploring and identifying the characteristics and behavioural traits of AHPs in clinical academic training and established roles.

## Methods

### General overview

This qualitative study was conducted using in-depth semi-structured interviews of clinical academic AHPs working in the UK. A phenomenological approach was used to explore the individual characteristics and behavioural traits of clinical academic AHPs through reflexive thematic analysis. The study is reported in accordance with the Consolidated criteria for reporting qualitative studies (COREQ), with a checklist available in supplementary material 1 [18].

### Participants

Participants were eligible to take part if they were established or aspiring clinical academic AHPs. Established clinical academic AHPs were defined as post-doctorate and employed in a combined clinical and research role with at least 25% of their contracted hours dedicated to clinical or research work. The threshold of doctoral qualification was chosen to define established clinical academic AHPs since this is necessary for leading grant submission, supervising doctoral students, and progressing into post-doctoral senior clinical academic roles. Aspiring clinical academic AHPs were defined as those at pre-doctoral stage in their research training and/or experience and wishing to pursue a clinical academic career. Participants were selected using a purposive sampling strategy with two approaches. First, the study was advertised using social media aiming to recruit aspiring clinical academic AHPs. Second, since the number of established clinical academic AHPs in the UK is relatively low, these individuals were contacted directly by email with an invitation to participate. These participants were selected by profiling academic institutions and AHP professional and research networks with the aim of ensuring invitations were extended to representatives of all fourteen AHP professions.

### Data collection

We conducted in-depth semi-structured online interviews to explore the individual character traits and behaviours of aspiring and established clinical academic AHPs. All authors (EK, TC, OG) developed the interview topic guide (supplementary material 2). The authors are all aspiring clinical academic AHPs who have previously undertaken research on the topic of clinical academia [6]. Both the individual experiences of the authors in developing a clinical academic career and their previous research informed the assumption that clinical academic careers for AHPs are challenging. Additionally, it was assumed that participants might too easily externalise challenges rather than explore what traits, behaviours or strategies they used to overcome them. These assumptions informed the development of the topic guide which was finalised after a pilot interview was conducted with an expert qualitative clinical researcher.

We contacted participants prior to the interview to establish a convenient time to undertake the interviews. Interviews were conducted via the videoconferencing platform Zoom, with audio recording only, and subsequently transcribed verbatim. Field notes were made during the interviews. All interviews were conducted by EK, who is a female clinical academic physiotherapist with experience of undertaking semi-structured interviews and is currently undertaking a qualitative PhD. EK had no previous working relationship or prior research collaborations with any of the study participants.

### Data analysis

The data were analysed using reflexive thematic analysis as per Braun and Clarke [19]. Reflexive thematic analysis allows for the creation of themes, which are patterns of shared meaning united by a central concept [20]. It is considered an active process as the codes and then the themes are developed. The analysis followed six phases. In phase one (familiarisation with the data) we listened to the audio recordings and read through the transcripts. Analysis proceeded to phase two where we closely examined and collaboratively coded the transcripts, which was undertaken manually without software. We then developed the themes from the codes, prior to further refining and finalising the themes (phases three, four and five). Finally, we produced and refined a report of the themes (phase six).

*Ethical considerations.*

Ethical approval was obtained from the university's research ethics committee (reg no.221565). Participants were provided with the participant information sheet and given an opportunity to ask questions prior to giving informed consent.

## Results

**Fig. 1:**
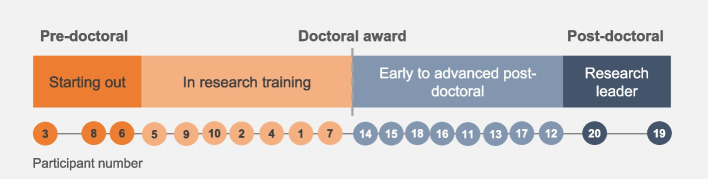
Participant clinical academic experience

Twenty participants from six allied health professions took part in the study. There were 15 (75%) females and five (25%) males, with a wide range of clinical academic experience. Table 1 outlines the self-reported participant characteristics which have been pooled to preserve anonymity. Figure 1 demonstrates the spread of clinical academic experience amongst participants. The level of experience ranges from those just starting out having undertaken one or two research projects to those who hold clinical academic professorial roles. Ten participants had completed their doctoral studies. The interviews were conducted between June and August 2022 and lasted between 22 and 58 min. One participant consented to be interviewed but was unable to take part due to illness.

**Table 1:** Participant characteristics

Characteristics		n	%
**Sex**	Female	15	75
	Male	5	25
**Self-reported ethnicity**	White-British	16	80
	White-European	1	5
	White-American	1	5
	Asian-British	1	5
	Mixed-British	1	5
**Experience**	Starting out	3	15
	In research training	7	35
	Early to Advanced post-doctoral	8	40
	Research leader	2	10
**Profession**	Dietitian	2	10
	Occupational Therapist	2	10
	Physiotherapist	11	55
	Podiatrist	1	5
	Radiographer	2	10
	Speech and Language Therapist	2	10

We developed five themes from 12 parent and 85 child codes. The themes developed are: risk and reward, don’t wait to be invited, shifting motivations, research is a team sport; and staying the course.

### Risk and reward

Risk taking was identified as a critical factor in the successful pursuit of clinical academia. Many of the participants cited the need to embrace risk to reap the potential rewards.

*P8: ‘And you have to be someone that's willing to get it wrong…. but equally, you have to be able to learn from those mistakes’*

This was particularly apparent in the early stages of research training and skill acquisition. Participants describe venturing outside their comfort zone and feeling anxious with the differential between their status as experienced and highly skilled clinicians, but complete novice researchers. Some felt they were starting over again trying to learn a new discipline, whilst less experienced clinicians were daunted by the prospect of a ‘dual training’ load as they tried to develop their clinical and research skills simultaneously.

*P16: ‘By the time we become clinical academics we've been highly skilled clinical people for maybe a couple of decades, or maybe longer… then we are dumped into this world where we now have to write and present and assess and review and all that and actually we're real we're right back to the start and that that comes quite high, and I think that for can be quite hard.’*

In pursuing a clinical academic career and working across the clinical and academic settings, the feeling of ‘imposter syndrome’ was common amongst participants. This was associated with insecurities arising from the differences in working cultures, attitudes, and the conflicting demands. One participant identified the importance of establishing credibility in both aspects of the clinical and academic role.

*P13: ‘I think we all have inherently imposter syndrome…’*

*P14: ‘I think about being a clinical academic, which is about not being in any one camp and just being an outsider but a credible outsider.’*

The uncertain nature of clinical academia such as securing roles, contract renewal, project funding, or pending the outcome of grant submissions was identified as a challenging aspect of the role. Several participants highlighted the need to tolerate these uncertainties and deploy a strategic and ‘long game’ approach, such as accepting short term contracts. Some participants accepted the uncertainty and seemingly enjoyed the competitive element of securing funding adopting a tactical approach to their workload management. This strategy may serve to dampen the stresses and perhaps compartmentalise the activity.

*P14: ‘I approach it all like somewhat of a game…’*

Established clinical academics who led research units reported a clear burden of responsibility in supporting novice researchers and sustaining a research team. This included the pressure that derives from investing time and effort to secure research grants as the necessary investment to employ research staff and deliver against their objectives.

*P19:’…so if I don't bring any grants in, then I don't have a team.’*

Some participants describe a fine line between success and failure in clinical academia with the need to gamble at high stakes with their precious time and resources to gain large research investment with no certainty of successful outcome.

*P20: ‘So we're at the final stage of it, and everybody felt confident we were going to get it, and then we didn't get it.’*

Others were happy to hedge their bets in seeking smaller funds and projects to better balance the risk and reward.

In summary, embracing risk was considered a necessity in pursuing clinical academia. The extent of risk taking, maintaining profile and credibility, and dealing with imposter syndrome were all evident challenges.

### Don’t wait to be invited

Pursuing a clinical academic career for many of the participants arose from being inquisitive, intuitive, and having an innate desire to learn more about clinical research. Irrespective of these early triggers, most describe creating their own opportunities rather than waiting for an invitation. The inquisitive nature described by participants seemed to stem from a problem-solving approach often catalysed by the uncertainties and knowledge gaps arising in clinical practice.

*P8: ‘... being inquisitive… wanting to understand why not just… but why that happens and why does that happen, and what can you do to improve it…’*

The role of intuition and instinct seem to drive a calling in some participants to ‘just get involved’. Of these, most did not see prior approval or permission in a professional context as a necessity.

*P1: ‘I think that's what a lot of people have done - sat there waiting [for opportunities] to appear, and I’m not one of these people to just sit back and wait for things to happen’*

*P9:’ …I’ve never sought approval from other people to do things, apart from the people who really matter in my life…’*

A minority of participants were more forthright in seeking new experiences and opportunities advocating a, ‘you’ve got to be in it to win it’ mentality.

*P10: ‘What I would never do is self-exclude so I always throw my hat in the ring…’*

In summary, inquisitive, intuitive, and instinctive traits seemed to play a fundamental role in generating new opportunities and experiences; seeking permission or approval to engage was rarely considered.

### Shifting motivations

A range of motivational factors were reported as the reasons participants decided to embark on a clinical academic career. Many identified reaching a point in their career of low stimulation, boredom or lack of variety found in an exclusively clinical role.

*P13: ‘... it's about challenging the status quo; it's not being afraid of change…’*

Some identified the drive to develop clinical services through enhancing the underpinning evidence base on which they were originally designed. Improving patient care as the primary driver was cited by some participants with a few going on to describe their aspirations to influence and change outcomes for broader clinical populations.

*P2: ‘...to discover the better option or whether there are other options, or whether we can approach things differently…and then overall we are improving the outcomes for the patients’*

*P11: ‘I can have a much wider influence and it's not just that patient in front of me …has a big impact that then changes the way that other physios look after their patients then even more patients will get benefit….’*

For those in well-established clinical academic careers, mentoring early career researchers was considered a primary motivation at this point in their career compared to an earlier stage when it was driven by undertaking primary research.

*P19:’…I think now it's more about bringing on the next generation, rather than doing primary research for myself...’*

This shift in motivation is likely to be linked to the desire to impart their wealth of experience and the responsibilities that come with being a senior academic and leader of research activity. Conversely, this responsibility weighed heavily on some participants in the form of pressure to perform and feelings of guilt where they fell short of their own expectations. Some cited adopting greater personal motivation and solace through their own research projects to manage their health and well-being when things became too challenging.

*P9: ‘I think at the moment my research is a bit of a safe haven for me…’*

Most participants described being motivated by their passion for research, improving practice, and strong work ethic whilst some sourced this more from personal achievements attained through indicators of research output and success. This latter motivational factor was present more in participants with less clinical academic experience.

In summary, motivation of clinical academic AHPs varies considerably but a consistent factor appears to be a desire to keep stimulated and change things for the better either in an altruistic or more individual sense.

### Research is a team sport

The importance of forming and sustaining effective teams and relationships was described by most participants. For some, these relationships, particularly in a mentorship context, represent defining moments in the success of their career. The value of a good mentor and/or source of early career support appeared to be reflected in the longevity of the relationship.

*P12: ‘…I've been doing this for 20 years or so, I've kept the same mentor because she worked so well for me personally and academically.’*

*P14: ‘Sure I'm still in touch with all of those early people… as the years have gone on I've developed my own different interests, different areas of specialty so I’ve actually just moved away from their topic areas. But they're still all always there…’*

Some participants recognised their mentor as a source of inspiration not just because of the support and advice, but through the behaviour modelled by the mentor.

*P5: ‘I think I've been very fortunate in having someone there who's quite driven and who's able to kind of bring me along in that journey as such.’*

Participants recognised the value and importance of a research team when compared to the struggle of facing the challenges alone. Although relationships were described as vulnerable to stress ahead of deadlines, camaraderie and support were described as a positive feature of the team environment.

*P8: And certainly, I couldn't do it myself, and if it wasn't for the rest of the team, I would really struggle…’*

Building networks and collaborations was seen as beneficial by most participants with a few citing a loss of control of their research from these relationships. Effective approaches described to facilitate these collaborations included open communication, flexibility of views, and fair distribution of the workload.

*P19: And it's about being able to form collaborative relationships. Sometimes you're the one giving, sometimes you're the one receiving them…, and you sort of nurture those over the years…’*

More experienced participants recognised the engagement of key non-researcher stakeholders, such as research and development managers and finance departments, as important enabling functions, which are often overlooked in relationship building.

In summary, building effective teams, relationships, and networks were recognised as essential, and sometimes career changing, interventions with great emphasis placed on the value of mentorship.

### Staying the course

In pursuing a clinical academic career, most participants describe characteristics of tenacity, dedication, and high work ethic.

As a highly competitive field with limited opportunities, participants highlighted the importance of being open-minded and setting realistic expectations to deal with frequent criticism and rejection. Increasing experience and depersonalising critique were outlined as important factors in processing and dealing with failure.

*P9: ‘…it's not easy, it's not easy, and I certainly did not go into it with stars in my eyes.’*

*P13: ‘It’s a case of learning, taking on board the feedback. Initially it feels very harsh, very critical because you put your heart and soul into developing this piece of work. But coming back a few weeks later and seeing where the gaps were and perhaps what you've missed, how you could improve things and using that as a tool to take things forward looking.’*

Most participants identified various ways in which they responded to failure and rejection, including taking time to accept the feedback, seeing it as an inevitable part of the industry, and as an opportunity to improve.

*P6: ‘I think it was sort of one of those things, I sort of said I had a little pity party for one, for one day and then thought, brush myself off and get on with it…’*

The need for insight into the highs and lows of academia and being persistent in a project you believe in was highlighted as an important way to effectively navigate the emotional toil.

*P12: ‘...when things are going really well you know you’re coming in for a big fall…it's just peaks and troughs and you just accept that's part of it…’*

Most participants described working considerably beyond their contracted hours, especially prior to deadlines for grant applications.

*P4: ‘... working that much…you know it does obviously have an impact at home.’*

Participants acknowledged the adverse impact this had on their personal lives and were grateful for family support. The need to balance work and personal life commitments was recognised by participants, who felt this was challenging to achieve whilst seeking a successful clinical academic career.

In summary, the pursuit of a clinical academic career can be tumultuous, including the need to frequently manage failure and rejection, as well as working beyond contracted hours. How the participants responded to failure and pressure appears to be key to career success and longevity.

## Discussion

Our study explored the character traits and behaviours of aspiring and established clinical academic AHPs. The themes we developed from our interviews indicate traits of high self-efficacy, opportunism, risk-embracing, and high motivation among clinical academic AHPs. The high levels of risk, uncertainty, and personal life compromise tolerated by clinical academic AHPs appears mainly as a response to the demands from the academic component of the role.

### Determinism

An explicit determination to succeed in clinical academia was apparent in all study participants, regardless of their experience. This manifested as traits of high self-efficacy such as inquisitiveness, opportunism, risk tolerance, high motivation, and resilience [21]. The concept of self-efficacy derives from social cognitive theory and considers the individuals’ belief in their abilities and actions to be a critical determinant of their success [22]. This sense of agency and empowerment within our cohort appeared to be driven by motivation to solve problems in clinical practice, a passion for conducting research, and to a lesser extent, personal reward. The causal relationship between high self-efficacy and academic success has been previously established, although not in clinical academic AHP roles [23]. However, consideration must be given to the role of mitigating and, at times, compromising behaviours in pursuing that success. Participants described working excessive amounts of unpaid hours, particularly in response to deadlines for project and grant submissions. Participants with more experience indicated their willingness to ‘go above and beyond’ derived from tenacious and persistent traits. Similar findings were reported of physicians in the context of clinical academic career development [24].

The extent to which this determinism was driven extrinsically by the demands of clinical academia is well illustrated by the description that personal life compromises were seen as an inevitable condition of success. Research exploring the traits and identity of clinical academic physicians concluded that few other roles create such an expectation not only of knowledge and skill acquisition, but in the strong personal, affiliative, and ethical values needed to be successful [25]. The reliance on mentorship as a source of support was highlighted by participants as a way of managing these high demands. For some, this took the form of long-standing relationships with early career mentors they considered role models and confidants. Mentorship capacity and capability in clinical research and academia is cited as a crucial intervention in realising success [26, 27].

### Response to rejection and failure

Rejection and failure are prominent and persistent features of academic work life [28]. For those pursuing a clinical academic career, accepting this as an inevitable consequence of the industry and using it for professional growth is a key finding from our study. This notion is supported in ‘a manifesto for failure’, which proposes three concepts important in the management of academic rejection: depersonalising, collectivising, and embracing [29]. To depersonalise in this context is to divest the research work of personal identity and emotion [30]. This idea is not to dissociate completely, but instead to levy the criticism and rejection against the work product rather than it be seen as an infliction of personal inadequacy of the producer. This is important since the misappropriation of workplace rejection as interpersonal can result in a disproportionate emotional response leading to stress, anxiety, and longer-term mental health issues [31]. A reflective cooling off period away from the activity was practised by some of our participants before reengaging and reframing the rejection as an opportunity to improve. The use of self-differentiation in this way can help to distinguish emotions immediately arising from the rejection from thoughts more reflective of the individuals’ personal values and beliefs in a non-emotional state. This is a way of gaining some internal validity to mediate a proportionate emotional response [32].

### Risk, tolerance for vulnerability or uncertainty

The high tolerance of risk and vulnerability was identified as a key behavioural strategy in our findings. Upon entering clinical academia, AHPs felt their status as senior clinicians was diluted with the newly acquired label of ‘novice researcher’. This, combined with the unfamiliarity of academic conventions and culture, gave rise to imposter syndrome and a loss of confidence. AHPs are not alone in this; similar experiences have been reported by clinical academics in the medical and nursing professions [33, 34]. Imposter syndrome occurs when an individual doesn’t feel competent or worthy of the position or status they have gained [35]. This is thought to arise more in individuals who struggle to internalise their achievements and those with a predisposition for high self-criticism [36]. Strategies employed by our cohort to overcome this included recognising their novice status as a moderating factor for performance expectation. This alludes to transferring some accountability from the typical internalisation of imposter syndrome to one that recognises the role of organisations in their behaviours that may elicit this problem in individuals, such as exerting unrealistic expectations [37].

The short-term nature of clinical academic contracts and funding cycles was highlighted as an enduring source of stress and frustration. This stemmed from uncertainty surrounding personal contract renewal and, for more established clinical academics, generating sufficient funding to sustain their research activity and staff. Despite long being recognised as a problem, nationally agreed contractual arrangements for clinical academic AHPs working in England remain elusive [38]. Participants adopted a strategic approach to manage this issue, including hedging their bets by pursuing multiple sources of potential income with the hope of proportional success. This invariably carries a risk that the invested time and effort does not realise the return on investment. In the event of insufficient investment, some participants described conducting unfunded research to maintain their profile and research credibility. A case-controlled study demonstrated that research funded through grant awards was more likely to lead to additional peer-reviewed publications, and wider dissemination and impact [39]. This suggests that conducting unfunded research may lead to diminishing returns. This is perhaps an area where the difference between the clinical and academic parts of the role is most stark. Participants outline vastly higher pressures and performance expectations to maintain their academic contract compared to the relative security of their clinical job. The need for greater integration of clinical and academic role components has previously been identified and may help balance the responsibilities over the entirety of the role [40].

### Strengths and limitations

A key strength of our study is that we believe it to be the first to explore the character traits and behaviours of clinical academic AHPs. In doing this, we report novel insights and findings that may help prepare and support future clinical academic AHPs in their quest to succeed in this field.

The main limitation of our study is that only six of the 14 (40%) allied health professions were represented. We believe this resulted from an under-representation of clinical academic practitioners due to very small numbers of the registered workforce in some professions such as art, drama, and music therapy. Further, although considered a relatively large AHP profession, paramedics were not represented in this study, which may stem from a lack of formalised research and clinical academic roles as a proportion of that workforce [41].

## Conclusion

Clinical academic AHPs possess character traits of high self-efficacy, motivation, and determination in their ambitions to be successful in this field. The high-performance demands placed on AHPs derive largely from the academic component of the role necessitating risk embracing strategies to sustain the funding and expected activity. How clinical academic AHPs responded to rejection and failure was seen as key behaviour and skill to augment professional development and growth. Reported characteristics considered necessary by our participants, such as determinism, tolerance of risk and rejection, and working long hours under pressure may well be perceived negatively or act as a deterrent from this career path. Future research and improvement strategies should embrace these uncomfortable findings as the current reality for many AHPs and explore effective ways to reduce the over-reliance on individual ‘strength of character’ as a means to thrive in this field. To this end, we suggest an approach that addresses system and individual factors. Firstly, a framework that formalises and harmonises the structure, contractual arrangements, and operationalisation of clinical academic AHP roles may improve access, sustainability, and experience of this career path. Secondly, in the spirit of ‘nothing worth doing comes easy’, clinical academic practice should be proportionately challenging to augment knowledge and skill development, but programmes of comprehensive and pragmatic education, guidance, and support are needed to better prepare AHPs for these challenges.

### Supplementary Information

**Additional file 1. **COREQ (Consolidated criteria for reporting qualitative research) checklist.

**Additional file 2. **Interview topic guide.

## Data Availability

All data generated or analysed during this study are included in this published article [and its supplementary information files].

## Abbreviations

AHP: Allied health professions
NIHR ICAP: National institute of health research integrated clinical academic programme
UK: United Kingdom
COREQ: Consolidated criteria for reporting qualitative studies
