# Supplementary material for: Exploring individual character traits and behaviours of clinical academic allied health professionals: a qualitative study
Source: BMC Health Serv Res. 2023 Sep 23;23:1025. doi: 10.1186/s12913-023-10044-2 (PMC10517465; doi:10.1186/s12913-023-10044-2)
Supplement: Supplementary file 2 — Additional file 2. Interview topic guide. [file 12913_2023_10044_MOESM2_ESM.docx]

**Exploring individual character traits and behaviours of clinical academic allied health professionals: a qualitative study.**

**Topic guide**

| **Aim / Research Questions / Objectives** |
| --- |
| **Aim:**  To explore and understand the personal characteristics, behavioural traits, and career decisions in relation to becoming a successful clinical academic allied health professional. |
| **Objectives:**  1. To explore the career journey and the underpinning rationale for career path decisions taken by AHPs that led to a clinical academic career  2. To understand the extent to which undergraduate training, and clinical practice influenced the AHP for a clinical academic career  3. To identify the perceived barriers and challenges to a clinical academic career  4. To explore and identify the personal strategies and character traits used to overcome barriers to becoming a clinical academic  5. To determine the level of perceived effort in becoming a clinical academic AHP in relation to other AHP career paths |

**Demographics**

Can you tell me your profession?

Can you tell me your clinical academic split?

Are you happy to answer questions on your ethnicity and sex?

Please can you state your ethnicity.

Please can you state your sex.

**Successful**

1. Can you tell me about your current clinical academic role?

Prompts: job planning, university/NHS, responsibilities,

1. Can you tell me about the decisions you took in pursuing a clinical academic career?

Prompts: clinical and research milestones, decision making,

1. Can you describe the impact on you of any unsuccessful research applications, grants or publications?

Prompts: personal impact, professional impact,

1. Can you describe how you keep motivated to overcome unsuccessful outcomes, and what strategies you use to do this?

Prompts: depth of motivation, personal strategies, wider strategies

1. Can you describe what keeps you engaged with the enduring and rollercoaster nature of a clinical academic role?

Prompts: uncertainty, successes and disappointments, prolonged timeframe to successful role,

1. Can you describe how you approach unexpected challenges or last-minute deadlines that occur?

Prompts: motivation, time management, balance of work/life, strategies

1. Can you identify any personal characteristics that lend you to undertaking a clinical academic role compared to a more traditional AHP team lead or managerial role?

Prompts: perceived effort, character traits

**Aspiring**

1. Can you tell me about any research experience you have?

Prompts: journal clubs, study involvement, publications, posters/abstracts, internships

1. Can you tell me about your decision making as you work towards a clinical academic role?

Prompts: clinical and research milestones, decision making

1. Can you describe the impact on you of any unsuccessful applications, grants or publications?

Prompts: personal impact, professional impact

1. Can you describe what keeps you motivated to work towards a clinical academic role?

Prompts: depth of motivation, careers aims,

1. Can you describe any strategies you use to keep you motivated and trying to progress?

Prompts: personal strategies, team strategies,

1. Can you identify any personal characteristics that lend you to undertaking a clinical academic role compared to a more traditional clinical or managerial AHP role?

Prompts: perceived effort, character traits
